# An 18-subject EEG data collection using a visual-oddball task, designed for benchmarking algorithms and headset performance comparisons

**DOI:** 10.1016/j.dib.2017.11.032

**Published:** 2017-11-13

**Authors:** Kay Robbins, Kyung-min Su, W. David Hairston

**Affiliations:** a Department of Computer Science, University of Texas-San Antonio, San Antonio, TX 78249, USA; b Human Research and Engineering Directorate, US Army Research Laboratory, Aberdeen Proving Ground, MD 21005, USA

**Keywords:** EEG, Visually evoked potential, EEGLAB, EEG study format (ESS), Hierarchical event descriptor (HED) tags

## Abstract

This data note describes an 18-subject EEG (electroencephalogram) data collection from an experiment in which subjects performed a standard visual oddball task. Several research projects have used this data to test artifact detection, classification, transfer learning, EEG preprocessing, blink detection, and automated annotation algorithms. We are releasing the data in three formats to enable benchmarking of EEG algorithms in many areas. The data was acquired using a Biosemi Active 2 EEG headset and includes 64 channels of EEG, 4 channels of EOG (electrooculogram), and 2 mastoid reference channels.

**Specifications Table**

**Table:** 

**Subject area**	Biology
**More specific subject area**	Neuroscience
**Type of data**	EEG from 18 subjects performing a visual oddball task
**How data was acquired**	Biosemi Active2 EEG headset (72 channels).
**Data format**	Raw, annotated EEG in EEGLAB .set format as well 2 forms of processed data
**Experimental factors**	The original purpose of this data collection was to compare the performance of 4 commercial headsets while subjects were performing a number of standard tasks. This note describes a subset of the data for a single headset and task [1]
**Experimental features**	Subjects viewed images displayed at 2-second intervals and distinguished target oddball images from expected images.
**Data source location**	Army Research Laboratory, Aberdeen MD, USA.
**Data accessibility**	The data is provided via links to public repositories.

**Value of the data**

• The experiment uses a standard, well-understood task for a moderate number of subjects.
• This collection has been used as test data for a variety of published and in-progress studies including a comparison of EEG headset performance for different manufacturers [1]; EEG preprocessing algorithms [2]; EEG classification, active learning, BCI calibration, and transfer learning studies: [3], [4], [5], [6], [7]; dataset imbalance issues [8]; and automated EEG annotation [9] as well as several ongoing ERP/ERSP regression studies.
• Data provided in several standardized formats eases access and use for multiple purposes.
• The publication of this data will also allow users to benchmark their results for comparison with several of the published studies listed above.

## 1 Data

EEG was recorded from 18 subjects as part of a larger headset comparison study [1]. The voluntary, fully informed consent of the persons used in this research was obtained in written form. The document used to obtain informed consent was approved by the U.S. Army Research Laboratory's Institutional Review Board (IRB) in accordance with 32 CFR 219 and AR 70-25 and is in compliance with the Declaration of Helsinki. The study was reviewed and approved (approval # ARL 14-042) by the U.S. Army Research Laboratory's IRB before the study began. The anonymized data contains no personally identifiable information.

The data for each subject includes 64 channels of EEG in a standard 10–20 channel configuration, four channels of EOG, and 2 mastoid channels. All EEG data is provided in EEGLAB [10] .set file format and is designed to be read and processed in MATLAB. The data is hosted in three formats on NITRC (www.nitrc.org) under the project Visually Evoked Potential EEG as follows:

1) Raw data in ESS (EEG study format) containerized format [11]. ESS provides a directory structure and an XML file with collection metadata to facilitate large-scale data processing. Alternatively, users can access the individual files without using the ESS structure. The individual EEG files are in standard EEGLAB .set format. The events in the EEG structure are identified by event codes and also annotated using Hierarchical Event Descriptor (HED) tags [12]. The data is located at ftp://ftp.nitrc.org/home/groups/vep_eeg_raw/VEPESS.zip.
2) Cleaned EEG files (as a single directory of .set files). The raw EEG was cleaned using the PREP pipeline to remove line noise and to identify and interpolate bad channels [2]. The data was then subjected to additional ICA-based artifact removal using MARA (Multiple Artifact Rejection Algorithm) [13], [14]. EEGLAB's runica function was used to calculate the ICA components. The data is located at: ftp://ftp.nitrc.org/home/groups/vep_eeg_raw/VEP_PREP_ICA_VEP2_MARA.zip.
3) Labeled sliding window power features based on the cleaned data of step 2), used for training and testing of automated EEG annotation [9]. Cleaned data was filtered in 4 Hz sub-bands starting at 0.75, 4, 8, 12, 16, 20, 24, and 28 Hz, respectively. The data in each sub-band was normalized to have 0 mean and unit standard deviation in each channel. The channel data was then windowed using overlapping sliding windows of one second spaced 125 ms apart. Each feature corresponds to a vector of 4096 elements: 64 channels×8 sub-bands×8 consecutive subwindows. A feature vector starting at each possible sub-window is computed. Feature vectors are labeled based on whether there is a friend (event code 34), foe (event code 35) or no event in the first sub-window of the feature vector. The data is located at: ftp://ftp.nitrc.org/home/groups/vep_eeg_raw/VEP_PREP_ICA_VEP2_MARA_averagePower.zip.

## 2 Experimental design, materials and methods

The experiment used a standard visually evoked potential (VEP) oddball task. Eighteen subjects were presented with a sequence of two types of images: a US soldier (friend) and an enemy combatant (foe). The images were presented in random order at a frequency of approximately 0.5±0.1 Hz. Subjects were instructed to identify each image with a button press. The following table summarizes the events that are embedded in the data. Approximately 1/7 of the images were oddball (foe) images. The button press events were not exactly synchronized with the actual physical button presses but rather were inserted at fixed intervals after the image presentation at the end of the trial periods. Separate external data files containing the actual response times are available from the authors upon request. Table 1 summarizes the experiment's event codes.

**Table 1:** Event codes used in the VEP experiment.

**Codes**	**Description**
17	Sync triggers to validate the timing accuracy of the headsets and align external data files. (Ignore for analysis.)
33	Experiment begins.
34	Primary stimulus (“friend”) onset (presentation of non-target image).
35	Oddball stimulus (“foe”) onset (presentation of target image).
38	Subject button-press response was correct. This is determined AFTER the subject response, and is not time-locked to a subject's response latency, but a fixed period after stimulus. Note that some epochs do not have this code due to a lack of a response from the subject.
39	Subject button-press response was incorrect. See above for some additional description.
63	Experiment ends.

Subjects sat approximately 70 cm from a Dell P2410 monitor to view images of 152×375 pixels, presented for 150 ms with an inter-image spacing of 1650 to 2150 ms. Non-target images contained a American soldier, while target images contained a man wearing a headscarf and holding a weapon. The images were presented in three blocks of approximately 89 trials. Experiments were conducted at the Army Research Laboratory in Aberdeen Maryland in a sound shielded room.
